# Robust multi-objective metaheuristic optimization of Cr(III) electrodeposition on 42CrMo4 steel for energy-efficient wear resistance

**DOI:** 10.1038/s41598-026-70987-4

**Published:** 2026-09-14

**Authors:** Talhi Amar, Djeffal Selman, Ghoul Abdelhamid

**Affiliations:** 1 National Polytechnic School of Constantine, Constantine, Algeria; 2 Automatic Control, Department of Computer Science, Electrical and Space Engineering, Luleå University of Technology, Luleå, 97187 Sweden

**Keywords:** Trivalent chromium, 42CrMo4 steel, Multi-objective optimization, MOPSO, NSGA-II, Harris hawks optimization, Wear, Surface roughness, Specific energy consumption, Energy science and technology, Engineering, Materials science

## Abstract

Selecting a Cr(III) electrodeposition condition for 42CrMo4 steel is a conflicting engineering problem: the current–temperature region that improves surface finish does not necessarily minimize wear or electrical demand, while the preparation route that maximizes hardness can shift the preferred operating window. Here, this conflict is resolved through an AI-inspired robust multi-objective metaheuristic framework that converts the original experimental dataset into a five-objective decision problem coupling surface roughness, wear, specific energy consumption (SEC), Faradaic efficiency and preparation-level hardness. The measured current sweep at 40 $^{\circ }$C, temperature sweep at 50 A, Vickers microhardness data, Faradaic-efficiency trend and SEC trend are represented by shape-preserving cubic interpolation; roughness and wear are combined through an explicitly identified geometric reconstruction of the two orthogonal experimental sweeps because a full current–temperature factorial matrix was unavailable. Multi-objective particle swarm optimization (MOPSO), NSGA-II and multi-objective Harris hawks optimization (MOHHO) independently search the same decision space while every candidate is stress-tested under *± 2* A and $\pm 2~^{\circ }$C perturbations. Despite their different exploration mechanisms, all three algorithms converge toward a consistent favorable domain, yielding a consensus compromise near 53.6 A and 39.7 $^{\circ }$C with diamond polishing, equivalent to a mean cathodic current density of about 53.6 A dm$^{-2}$ for the 1.0 dm$^{2}$ plated specimen area. The corresponding robust reconstructed responses are approximately 0.112 *μ *m worst-case roughness, 4.29 mg worst-case wear and 91.3% minimum Faradaic efficiency, with a preparation-level mean hardness of about 1131 HV. Because the post-Pareto selector contains engineering preferences, this point is treated in the current manuscript as an equal-weight reference compromise rather than as a universal service optimum; explicit weight-priority and non-separable current–temperature interaction stress tests are reported below. The result solves the practical selection problem of choosing one defensible operating region when experimentally favorable conditions conflict across tribology, coating quality and process energy, while preserving a clear distinction between measured observations and post-experimental reconstructed responses.

## Introduction

Hard chromium remains an important functional coating for steel components exposed to sliding, repeated contact and corrosive environments. Conventional Cr(VI)-based plating, however, carries severe toxicological and regulatory concerns, which has motivated the development of trivalent chromium electrolytes as lower-hazard alternatives. Leimbach et al. used electrochemical measurements to show that near-surface pH and local cathodic chemistry strongly influence chromium deposition from Cr(III) solutions^1^. Song and Chin demonstrated that current efficiency is strongly affected by polarization and parasitic reactions, particularly hydrogen evolution^2^. Protsenko and Danilov further showed that complexing chemistry modifies the kinetic pathway of Cr(III) reduction^3^. These studies establish a central point for process optimization: the electrical setting, bath chemistry and interfacial state are coupled, and applied current should not be interpreted as an isolated quality variable.

The modern Cr(III) literature has increasingly shifted from replacement chemistry toward performance engineering. Liang et al. reported that a chromium coating deposited from a Cr(III) electrolyte can approach the anti-wear performance of conventional hard chromium when deposition conditions are properly controlled^4^. Mahdavi et al. linked Cr(III)-derived coating properties to processing conditions and coating characteristics^5^. Guillon et al. investigated the mechanical behaviour of hard chromium produced from a trivalent bath and highlighted the importance of structure-sensitive mechanical characterization^6^. More recently, Broitman et al. connected thin dense chromium microstructure with nanomechanical and tribological response^7^, while Protsenko reviewed the current state of eco-friendlier Cr(III) electrodeposition and emphasized the combined roles of bath composition, current density, additives and microstructure^8^.

Surface preparation adds another layer of coupling. Mechanical and electrolytic polishing alter asperity geometry, residual surface condition, activation behaviour and nucleation density before chromium deposition. Yildiz et al. reviewed surface treatments used to create wear- and corrosion-resistant steel surfaces and emphasized that preparation route is a functional design choice rather than a cosmetic finishing step^9^. Liu et al. likewise showed that mechanical polishing can modify friction and corrosion behaviour through changes in the pre-coating surface state^10^. For chromium coatings specifically, crack density and defect structure can influence tribological response^11^, and surface texturing or preparation can modify the wear of coated steels^12^.

Current density and temperature are equally influential. Tey et al. showed that Cr–C electrodeposition from a trivalent bath requires simultaneous control of current density, pH, bath temperature and time to obtain high hardness and corrosion resistance^13^. Hagarová et al. reported a strong current-density dependence of mechanical and tribological behaviour in wear-resistant chromium coatings^14^. Sherwin et al. reached a similar conclusion for Ni–Cr electrodeposition, where current-density selection changed morphology, grain refinement and cathodic efficiency^15^. Taken together, these results make a single “best current” or “best polishing route” scientifically incomplete because the preferred condition depends on which response is prioritized.

Wear introduces a further trade-off. Initial hardness and roughness do not uniquely determine sliding durability because the interface evolves through oxidation, debris formation, cracking and tribolayer development. Jia et al. demonstrated that structural relaxation and oxidation can modify nanoscopic wear behaviour^16^. Zakiev et al. connected micromechanical properties with friction and wear-track response for Cr, Mo and Ta coatings^17^. Alternating chromium architectures have also been proposed to improve wear resistance by redistributing localized damage^18^. These studies motivate a multi-response rather than single-response description of functional coating performance.

Recent work in extitScientific Reports has reinforced this optimization perspective: nature-inspired metaheuristics have been described as important components of artificial intelligence for difficult multidisciplinary optimization problems^19^, multi-objective metaheuristic search has been used to resolve conflicting materials-processing responses in electrical-discharge machining^20^, and response-surface optimization has recently been applied directly to jet electrodeposition while linking process settings to hardness and tribological performance^21^.

The previous form of the present 42CrMo4 study compared surface preparation, applied current, bath temperature, microhardness, roughness, wear, Faradaic efficiency and SEC within one experimental manuscript. That integrated experimental basis is retained here, but the optimization logic is fundamentally strengthened. Instead of declaring an optimum from visual inspection of individual curves, the measured trends are treated as the input to a robust Pareto problem solved independently by MOPSO, NSGA-II and MOHHO. The objective is not to force three algorithms to produce different operating points; rather, it is to determine whether independent search mechanisms identify a consistent favorable domain and to expose the trade-off structure among roughness, wear, energy demand, efficiency and hardness.

The main contributions are therefore: (i) a five-objective Cr(III) electrodeposition formulation using current, temperature and surface preparation as decision variables; (ii) a deterministic robustness test over 25 local current–temperature perturbations for every candidate; (iii) a transparent reconstruction of a two-dimensional response from the two available orthogonal experimental sweeps without presenting the reconstruction as new measurement; (iv) a three-algorithm Pareto comparison using MOPSO, NSGA-II and MOHHO; and (v) roughness-consistent reconstructed surface profiles and PSD representations used only for morphological interpretation. In the current manuscript, the metaheuristic results are additionally verified against deterministic baselines (an exhaustive fine grid search and an equal-weight weighted-sum scalarization), and explicit sensitivity checks of the reconstruction rule and of the hardness objective are reported in Sect. “Baseline comparisons and model-sensitivity checks”. The remainder of the paper is organized as follows. Section “Methods” presents the experimental basis and optimization formulation, Sect. “Results” reports the Pareto and reconstructed-profile results, Sect. “Discussion” discusses their physical meaning and limitations, and Sect. “Conclusion” summarizes the principal findings.

## Methods

### Material, surface preparation and experimental basis

The substrate is 42CrMo4 Cr–Mo low-alloy steel (Table 1). The mechanical and compositional values retained from the original experimental study are condensed in Table 2. The substrate was subjected to three surface-preparation routes for the numerical comparison used here: diamond polishing, 1200-grit mechanical polishing and electrolytic polishing. Cr(III) electrodeposition was then evaluated through two orthogonal parameter sweeps. The current sweep used *I=30,40,50,60,* and 70 A at $T=40~^{\circ }$C, while the temperature sweep used *T=30,35,40,45,50,55,* and $60~^{\circ }$C at *I=50* A. Surface roughness $R_a$ and wear loss *W* were available for each preparation route in both sweeps. Faradaic-efficiency and SEC trends were available as functions of applied current, and Vickers hardness was measured at indentation loads from 25 to 1000 g.

The substrates were rectangular plates of $100\times 50\times 5$ mm machined from a single normalized 42CrMo4 bar. The two principal faces were active during deposition while the edges were masked with a chemically resistant lacquer, giving an effective plated area of $A_{\textrm{eff}}\approx 1.0$ dm$^{2}$ per specimen. Every applied current can therefore be converted into a mean cathodic current density through $J=I/A_{\textrm{eff}}$, so the investigated range of 30–70 A corresponds numerically to 30–70 A dm$^{-2}$, and the consensus compromise discussed in Sect. “Results” corresponds to about 53.6 A dm$^{-2}$. Reporting both quantities allows the identified operating window to be transferred to workpieces of different size, since the intrinsic electrochemical variable is the current density rather than the machine current setting. The mean coating thickness at the reference condition of 50 A and 40 $^{\circ }$C was *20± 3* *μ *m, determined from cross-sectional optical micrographs at five positions along the plate.

The three surface-preparation routes were applied under fixed, documented conditions, which are summarized in Table 1. All specimens were first ground with successive SiC papers under water lubrication. The diamond route continued with 6 *μ *m and then 1 *μ *m diamond suspensions on woven cloths; the 1200-grit route stopped after the final SiC stage; the electrolytic route was ground to 800-grit and then electropolished in a phosphoric–sulfuric acid mixture. Before deposition, every specimen was ultrasonically degreased in acetone, alkaline cleaned, activated in diluted hydrochloric acid and rinsed in distilled water. The resulting pre-deposition roughness of each route is included in Table 1; these starting values define the reference surface state on which the coating-level roughness discussed in Sect. “Results” develops.

**Table 1:** Surface-preparation parameters and pre-deposition roughness of the three routes. All mechanical stages were water- or suspension-lubricated; the pre-deposition $R_a$ is the mean ± one standard deviation of five stylus traces per route.

Route	Final finishing stage	Duration of final stage	Operating conditions	Pre-deposition $R_a$ [*μ *m]
Diamond	6 *μ *m then 1 *μ *m diamond suspension on woven cloth, after SiC grinding P320–P1200	5 min per suspension	Rotary disc, 150 rpm, *≈ *15 N normal load	*0.045± 0.006*
1200-grit	SiC grinding P320*→ *P1200, water lubricated	4 min on final paper	Rotary disc, 250 rpm	*0.105± 0.012*
Electrolytic	Electropolishing after P800 grinding	4 min	H$_3$PO$_4$:H$_2$SO$_4$ 60:40 vol., 50 $^{\circ }$C, 15 A dm$^{-2}$, stainless-steel cathode	*0.078± 0.010*
Common pre-treatment	Ultrasonic acetone degreasing (10 min); alkaline cleaning; activation in 10 vol.% HCl (30 s); distilled-water rinse	–	Room temperature	–

Measurement repetition and uncertainty are reported as follows. Each roughness value is the mean of five stylus traces taken at different positions and orientations on the coated face, with a typical standard deviation below 8% of the mean. Each wear value is the mass loss of one tribological test, obtained as the difference of specimen masses averaged over three successive weighings on an analytical balance with 0.1 mg resolution. Vickers hardness at every indentation load is the mean of five indentations, with a scatter of about *± 4*%. The Faradaic-efficiency and SEC values derive from one deposition series per current level; they are therefore treated as comparative laboratory trends rather than replicated population estimates, and this limitation is restated in Sect. “Discussion” together with the planned replicated factorial campaign.

**Table 2:** Condensed 42CrMo4 substrate and experimental basis. The electrical axis is reported as applied current; with the effective plated area of 1.0 dm$^{2}$ described above, the same numerical values correspond to the mean cathodic current density in A dm$^{-2}$..

Category	Parameter	Value / levels
Mechanical	Hardness; yield strength; tensile strength	270–330 HB; 650 MPa; 900–1100 MPa
Mechanical	Elongation; impact resilience	$\approx 12\%$; 41 J cm$^{-2}$
Composition	C; Si; Mn	0.38–0.45; 0.15–0.40; 0.50–0.80 wt.%
Composition	P; S; Cr; Mo	*≤ 0.035*; *≤ 0.035*; 0.90–1.20; 0.15–0.30 wt.%
Preparation	Surface routes	Diamond; 1200-grit; electrolytic polishing
Current sweep	*I* at fixed $T=40~^{\circ }$C	30, 40, 50, 60, 70 A
Temperature sweep	*T* at fixed *I=50* A	30, 35, 40, 45, 50, 55, 60 $^{\circ }$C
Hardness	Vickers indentation load	25, 50, 100, 200, 300, 500, 1000 g

**Fig. 1:**
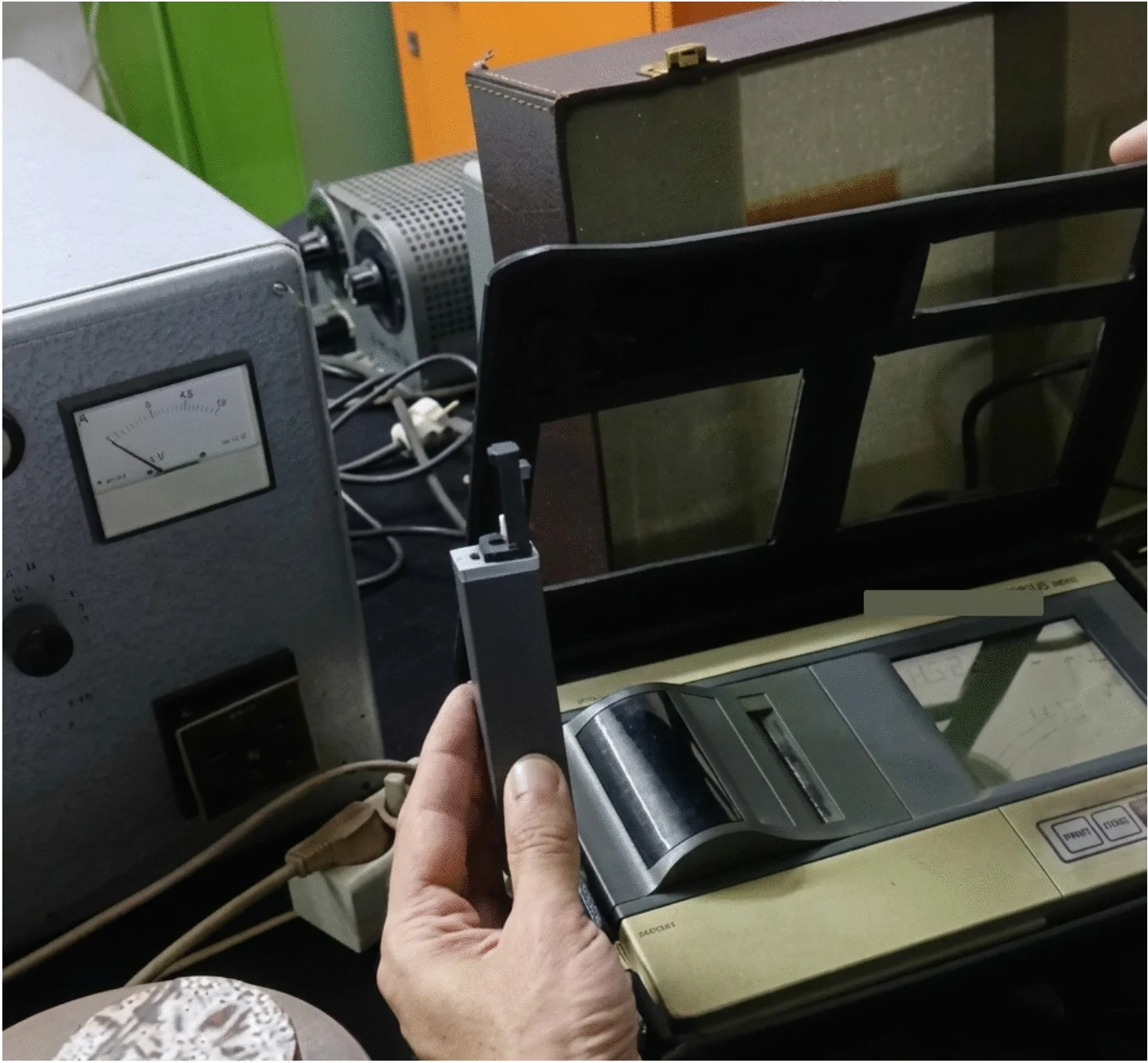
Microhardness measurement arrangement used for the chromium-coated specimens. The physical test stage provides the preparation-resolved hardness data later condensed into the hardness objective of the multi-objective problem. The laboratory photograph is intentionally shown without manufacturer branding because such identification is not required for the technical interpretation of the setup.

The microhardness measurement arrangement retained from the original work is shown in Fig. 1. This experimental image is included to distinguish the physical characterization stage from the later post-experimental numerical reconstruction.

### Electrochemical efficiency indicators

The theoretical chromium mass associated with an applied current follows Faraday’s law,

1 $$\begin{aligned} m_{\textrm{th}}=\frac{MIt}{nF}, \end{aligned}$$

where *M* is the molar mass of chromium, *I* is applied current, *t* is deposition time, *n=3* is the electron number for Cr(III) reduction and *F* is Faraday’s constant. The cathodic or Faradaic efficiency is

2 $$\begin{aligned} \eta _F=100\frac{m_{\textrm{exp}}}{m_{\textrm{th}}}. \end{aligned}$$

The corresponding specific energy consumption can be written as

3 $$\begin{aligned} \textrm{SEC}=\frac{U_{\textrm{cell}}It}{m_{\textrm{exp}}}, \end{aligned}$$

with the appropriate conversion factor when expressed in kWh kg$^{-1}$. In the supplied dataset, the available SEC values are a current-dependent comparative series rather than a complete industrial energy audit because continuous cell-voltage logging and the full raw deposited-mass records were not included. The values are therefore retained for relative optimization but are not interpreted as a validated lifecycle-energy balance.

It should be emphasized that the SEC defined in Eq. (3) is normalized by the deposited chromium mass, so every value in the series is an energy demand per unit mass of coating (kWh kg$^{-1}$) and is directly comparable across current levels without further scaling. Because the effective plated area is now reported in Sect. “Material, surface preparation and experimental basis”, the same series can also be re-expressed per unit coated area once a target coating thickness is specified, which is the natural form for scale-up estimates. What the series does not capture are plant-level contributions such as bath heating, agitation and rectifier losses; for this reason, the SEC term enters the optimization as a relative penalty that ranks operating currents, and the absolute values should not be quoted as a production energy footprint.

### Response interpolation and orthogonal-sweep reconstruction

Shape-preserving piecewise cubic Hermite interpolation was used between the discrete experimental levels. This prevents the optimizer from being restricted to the five measured current settings or seven temperature settings while avoiding the oscillatory behaviour that can arise from unconstrained high-order polynomials. The experimental design did not contain a full factorial *I× T* matrix. Consequently, no claim is made that the two-dimensional surfaces in this study were directly measured.

For roughness and wear, the current and temperature sweeps were fused through a geometric reconstruction. For preparation route *P*,

4 $$\begin{aligned} \widehat{R}_a(I,T,P)=\sqrt{R_{a,I}(I,P)R_{a,T}(T,P)}, \end{aligned}$$

and

5 $$\begin{aligned} \widehat{W}(I,T,P)=\sqrt{W_I(I,P)W_T(T,P)}. \end{aligned}$$

This positive-valued fusion preserves the order of magnitude of both orthogonal sweeps and creates a continuous surrogate response for optimization. Equations (4)–(5) are computational reconstructions, not substitute experimental measurements.

Two properties of the geometric fusion can be checked directly against the data. First, the two sweeps share the anchor condition of 50 A and 40 $^{\circ }$C, at which every response was measured twice, once in each sweep. At this common point the reconstruction returns the geometric mean of the duplicated measurements: for wear, the two independent values agree within 2.3–5.6% for the three preparation routes (4.30/4.40 mg for diamond, 2.10/2.20 mg for 1200-grit and 1.70/1.80 mg for electrolytic polishing), and for roughness, the 1200-grit pair agrees within 2.9% (0.068/0.070 *μ *m), while the diamond and electrolytic pairs differ by about 38% (0.130/0.080 and 0.080/0.130 *μ *m), which mainly reflects replicate scatter of low-$R_a$ stylus measurements rather than a failure of the fusion itself. Second, along the two measured lines the reconstruction reproduces the shape of each experimental sweep exactly, up to a positive scale factor, so no measured trend is distorted. The fusion remains a separable first-order approximation: it cannot generate current–temperature interaction terms that were never sampled. To quantify how strongly the conclusions depend on this choice, Sect. “Baseline comparisons and model-sensitivity checks” repeats the complete robust optimization with two alternative fusion rules (arithmetic mean and elementwise maximum); the compromise moves by at most 2.0 A and 1.7 $^{\circ }$C and never changes the selected preparation route. The definitive assessment can nevertheless only come from a factorial *I× T* campaign, which is planned around the identified basin as described in Sect. “Discussion”.

The alternative fusion rules above are still separable, so they do not by themselves answer the stronger question of an unknown electrochemical *I*–*T* interaction. We therefore added a deliberately non-separable interaction-envelope stress test. For $y\in \{R_a,W\}$, the separable reconstruction $\widehat{y}_0$ is perturbed as

6 $$\begin{aligned} \widehat{y}_{\kappa ,c}(I,T,P)=\widehat{y}_0(I,T,P)\left[ 1+c\,g(I,T)\right] ,\qquad g(I,T)=\tanh \!\left( \frac{I-50}{5}\right) \tanh \!\left( \frac{T-40}{2}\right) , \end{aligned}$$

with $c\in [-\kappa ,+\kappa ]$. This construction is intentionally a stress-test envelope rather than a fitted electrochemical law. It is useful because *g=0* on both experimentally sampled axes (*I=50* A and $T=40~^{\circ }$C), so all measurements in the two original sweeps remain exactly unchanged, whereas away from those axes the model contains a genuine cross term and a current perturbation can have a different effect above and below 40 $^{\circ }$C. During robust evaluation the worst sign of *c* is included together with the 25 $(\Delta I,\Delta T)$ combinations. Two deliberately large envelopes, *κ =0.25* and 0.50, corresponding to at most 25% and 50% multiplicative interaction modulation away from the measured axes, are examined in Sect. “Baseline comparisons and model-sensitivity checks”. These values are not claimed as measured interaction magnitudes; they are used to test whether the reported decision domain collapses when separability is explicitly broken.

### Robust five-objective formulation

The decision vector is

7 $$\begin{aligned} \textbf{x}=[I,T,P], \end{aligned}$$

with $30\le I\le 70$ A, $30\le T\le 60~^{\circ }$C and $P\in \{1,2,3\}$ denoting diamond, 1200-grit and electrolytic preparation, respectively. To avoid selecting a narrow optimum that is favorable only at one exact operating point, each candidate is evaluated on the local perturbation set

8 $$\begin{aligned} \Delta I,\Delta T\in \{-2,-1,0,1,2\}, \end{aligned}$$

which yields 25 current–temperature combinations around every nominal candidate.

The perturbation amplitudes in Eq. (8) were chosen from the stability of the laboratory equipment rather than arbitrarily. The plating rectifier holds the current setpoint within about *± 1*% of reading, i.e. at most *± 0.7* A over the investigated range, and the thermostatically controlled bath shows short-term temperature fluctuations of about $\pm 1~^{\circ }$C with spatial gradients of up to roughly 1.5 $^{\circ }$C between the heater and the specimen zone. The values *± 2* A and $\pm 2~^{\circ }$C therefore represent a deliberately conservative envelope of roughly twice the observed short-term variability, so that a candidate is retained only if it stays acceptable under drifts larger than those expected in routine operation. Concerning the density of the check, the *5× 5* grid is not an arbitrary truncation: the objective surfaces are built from shape-preserving piecewise-cubic interpolants of coarsely spaced measurements, so they contain no oscillation finer than the experimental spacing, and the neighbourhood worst case is attained on or very close to the perturbation-box boundary. Repeating the evaluation at the consensus point with a dense *81× 81* neighbourhood grid (0.05 A *× * $0.05~^{\circ }$C spacing) changes the four robust objectives by less than 0.16% for every preparation route, which confirms that the 25-point grid captures the neighbourhood worst case at negligible computational cost.

The five minimization objectives are

9 $$\begin{aligned} f_1&= \max _{\Delta I,\Delta T}\widehat{R}_a(I+\Delta I,T+\Delta T,P),\end{aligned}$$

10 $$\begin{aligned} f_2&= \max _{\Delta I,\Delta T}\widehat{W}(I+\Delta I,T+\Delta T,P),\end{aligned}$$

11 $$\begin{aligned} f_3&= \max _{\Delta I}\textrm{SEC}(I+\Delta I),\end{aligned}$$

12 $$\begin{aligned} f_4&=100-\min _{\Delta I}\eta _F(I+\Delta I),\end{aligned}$$

13 $$\begin{aligned} f_5&=\overline{HV}_{\max }-\overline{HV}(P). \end{aligned}$$

The hardness term uses the mean Vickers hardness across the available indentation loads for each preparation route because hardness was not available as a full current–temperature response surface. The preparation-level means are approximately 1131, 979 and 887 HV for diamond, 1200-grit and electrolytic polishing, respectively.

The hardness term $f_5$ deserves an explicit caveat. In trivalent chromium deposition, current density and bath temperature influence grain size, microcracking and residual stress, and therefore hardness^13–15^. In the available dataset, however, Vickers hardness was measured across indentation loads for the three preparation routes only, not as a current–temperature map, and constructing such a dependence numerically without measurements would amount to inventing data. The term $f_5$ is therefore a preparation-level hardness index whose role is to discriminate between the categorical routes, not a deposition-condition-resolved hardness model. The consequence of this simplification is quantified in Sect. “Baseline comparisons and model-sensitivity checks”: when $f_5$ is removed from the formulation entirely, the compromise migrates to the electrolytic route near 50.2 A and 50.4 $^{\circ }$C, which shows that the recommendation of diamond polishing rests on the measured preparation-level hardness advantage and must be re-examined once condition-resolved hardness data around the Pareto basin become available.

### Metaheuristic search and post-Pareto compromise selection

Three independent population-based strategies were implemented directly in MATLAB: MOPSO, NSGA-II and a multi-objective Harris hawks optimizer. MOPSO extends the particle-swarm concept of Kennedy and Eberhart^22^ with an external non-dominated archive. NSGA-II uses non-dominated sorting and crowding distance to preserve Pareto diversity^23^. MOHHO adapts the exploration–exploitation mechanism of Harris hawks optimization^24^ to archive-based leader selection. All methods optimize Pareto dominance directly; no weighted objective is used during the search.

The reported figure set was generated with eight independent runs per algorithm, population size 60, 90 iterations and archive capacity 140. These values correspond to the computational package used to generate the supplied figures. Table 3 summarizes the formulation, while Algorithm 1 provides the complete computational sequence linking the reconstructed responses in Eqs. (4), (5), the robust perturbation set in Eq. (8), the five-objective evaluation in Eq. (13), and the post-Pareto compromise rule in Eq. (14). After optimization, a single engineering compromise is selected only for interpretation by minimizing the equal-weight normalized Euclidean distance to the utopia point,

14 $$\begin{aligned} D_u({\textbf {f}})=\sqrt{\frac{1}{5}\sum _{j=1}^{5}\left( \frac{f_j-f_j^{\min }}{f_j^{\max }-f_j^{\min }}\right) ^2}. \end{aligned}$$

The equal-weight choice in Eq. (14) is a preference-neutral reference rule, not a statement that a 1% physical change in hardness is equivalent to a 1% physical change in wear. Each term is first min–max normalized by its own attainable objective span; equal weighting therefore assigns equal importance to equal fractions of those normalized spans. Nevertheless, the selected route can legitimately change when the application values wear, hardness, energy or finish differently. To expose that dependence, the current manuscript additionally evaluates the weighted normalized distance

15 $$\begin{aligned} D_w({\textbf {f}})=\sqrt{\frac{\sum _{j=1}^{5} w_j z_j^2}{\sum _{j=1}^{5}w_j}},\qquad z_j=\frac{f_j-f_j^{\min }}{f_j^{\max }-f_j^{\min }},\quad w_j\ge 0, \end{aligned}$$

over the same deterministic 0.1 A *× * 0.1 $^{\circ }$C grid. We report balanced, wear-priority and hardness-de-emphasized scenarios rather than asserting one set of “typical” weights, because the correct utility weights depend on the component, contact regime and allowable coating failure mode. The resulting route switches are reported explicitly in Sect. “Baseline comparisons and model-sensitivity checks”; accordingly, the 53.6 A/39.7 $^{\circ }$C diamond point is referred to as the equal-weight reference compromise, not as a universal optimum. Thus, the scalar distance in Eq. (14) does not replace the multi-objective optimization; it is used only after the Pareto sets have been generated.

**Table 3:** Robust multi-objective formulation and common algorithm settings.

Item	Definition
Decision vector	$\textbf{x}=[I,T,P]$; *I∈ [30,70]* A; $T\in [30,60]~^{\circ }$C; *P=* diamond, 1200-grit or electrolytic
Objectives	Minimize worst-case reconstructed $R_a$, worst-case reconstructed wear, worst-case SEC, efficiency penalty $100-\eta _F$, and hardness penalty
Robustness test	$\Delta I=\{-2,-1,0,1,2\}$ A and $\Delta T=\{-2,-1,0,1,2\}~^{\circ }$C; 25 local combinations per candidate
Interpolation	Shape-preserving PCHIP between measured levels; geometric fusion of orthogonal *I* and *T* sweeps for $R_a$ and wear
Algorithms	MOPSO; NSGA-II; MOHHO
Reported settings	8 independent runs; population 60; 90 iterations; external archive up to 140 solutions
Final compromise	Minimum normalized distance to the utopia point after Pareto optimization; reported as an equal-weight reference and supplemented by weighted-priority sensitivity using Eq. (15)

**Algorithm 1:**
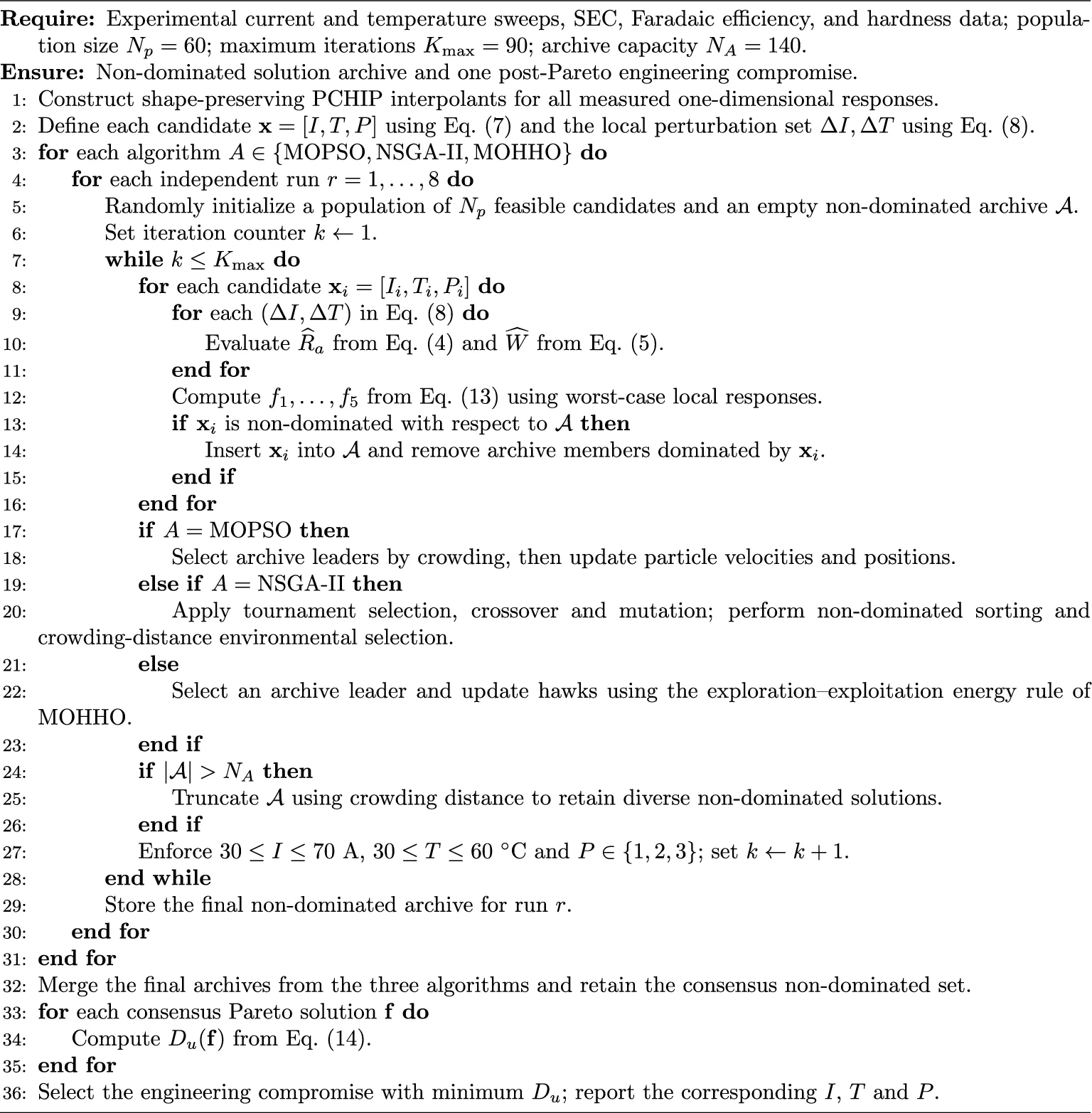
Unified robust multi-objective metaheuristic workflow for Cr(III) electrodeposition.

The pseudocode in Algorithm 1 emphasizes that the three optimizers share the same experimental response model and robust objective evaluation; only their population-update mechanisms differ. This common evaluation backbone allows the resulting Pareto distributions to be compared without changing the physical objective definitions between algorithms.

### Roughness-consistent reconstructed profiles and PSD

A final visualization layer was used to interpret the scale of surface fluctuations without presenting synthetic traces as profilometer measurements. For each preparation route, a deterministic multiscale profile was generated and then rescaled so that

16 $$\begin{aligned} R_a^{\textrm{profile}}=\frac{1}{N}\sum _{i=1}^{N}|z_i-\bar{z}|=R_a^{\textrm{target}}. \end{aligned}$$

At the selected operating condition, the target values were 0.0964 *μ *m for diamond polishing, 0.0706 *μ *m for 1200-grit polishing and 0.0998 *μ *m for electrolytic polishing. The PSD curves are derived directly from these reconstructed profiles by Welch averaging; no artificial spectral peaks are inserted.

In the current analysis, the reconstructed profiles are defined on a physical coordinate: each trace covers an evaluation length of 4 mm sampled at 1 *μ *m intervals (*N=4000* points), consistent with the ISO 4288 evaluation-length recommendation for the measured $R_a$ class. All profile axes are therefore expressed in millimetres and all spectral axes in cycles per millimetre. It must be stressed that this sampling convention is part of the stated reconstruction, in that it assigns a physically meaningful length scale to computational surrogate profiles; it does not replace stylus profilometry with a documented instrument pitch, which remains part of the planned experimental validation.

## Results

### Experimental trends motivating multi-objective optimization

The source measurements contain clear conflicts among the response variables. At 40 $^{\circ }$C, diamond polishing reaches its lowest roughness at 70 A ($R_a=0.078~\mu$m) but its lowest wear at 60 A (1.10 mg). The 1200-grit route reaches its minimum current-sweep roughness at 40 A (0.065 *μ *m) and minimum wear at the same current (2.00 mg). Electrolytic polishing reaches its minimum current-sweep roughness at 60 A (0.072 *μ *m), whereas its lowest current-sweep wear occurs at 70 A (1.35 mg). The temperature sweep introduces a different preference: at 50 A, 1200-grit polishing reaches 0.050 *μ *m at 50 $^{\circ }$C, diamond polishing reaches 0.062 *μ *m at 45 $^{\circ }$C, and electrolytic polishing reaches 0.060 *μ *m at 50 $^{\circ }$C. Wear favours electrolytic polishing much more strongly, reaching 0.10 mg at 30 $^{\circ }$C. The current-dependent energy series, by contrast, has its highest Faradaic efficiency (93%) and lowest SEC (7450 kWh kg$^{-1}$ in the supplied comparative series) at 50 A. These conflicting optima justify a Pareto treatment rather than selection from any single curve (Table 4).

**Table 4:** Condensed response minima and robust consensus result. Experimental minima are taken directly from the two supplied one-dimensional sweeps. The final row is a post-experimental robust Pareto result.

Condition / route	$R_a$ [*μ *m]	Setting	Wear [mg]	Setting	Efficiency / hardness	Energy indicator
Diamond, current sweep	0.078	70 A	1.10	60 A	mean 1131 HV	–
1200-grit, current sweep	0.065	40 A	2.00	40 A	mean 979 HV	–
Electrolytic, current sweep	0.072	60 A	1.35	70 A	mean 887 HV	–
Diamond, temperature sweep	0.062	45 $^{\circ }$C	4.00	30 $^{\circ }$C	–	–
1200-grit, temperature sweep	0.050	50 $^{\circ }$C	1.25	50 $^{\circ }$C	–	–
Electrolytic, temperature sweep	0.060	50 $^{\circ }$C	0.10	30 $^{\circ }$C	–	–
Current-energy anchor	–	50 A	–	–	$\eta _F=93\%$	SEC = 7450 kWh kg$^{-1}$
Robust Pareto compromise	0.112$^{\dagger }$	53.6 A, 39.7 $^{\circ }$C	4.29$^{\dagger }$	same	$\eta _F^{\min }\approx 91.3\%$; 1131 HV	SEC$^{\max }\approx 7613$ kWh kg$^{-1}$

$^{\dagger }$Worst-case reconstructed values over the *± 2* A/$\pm 2~^{\circ }$C perturbation neighbourhood; they are not additional physical measurements

### Pareto decision-space agreement across three algorithms

**Fig. 2:**
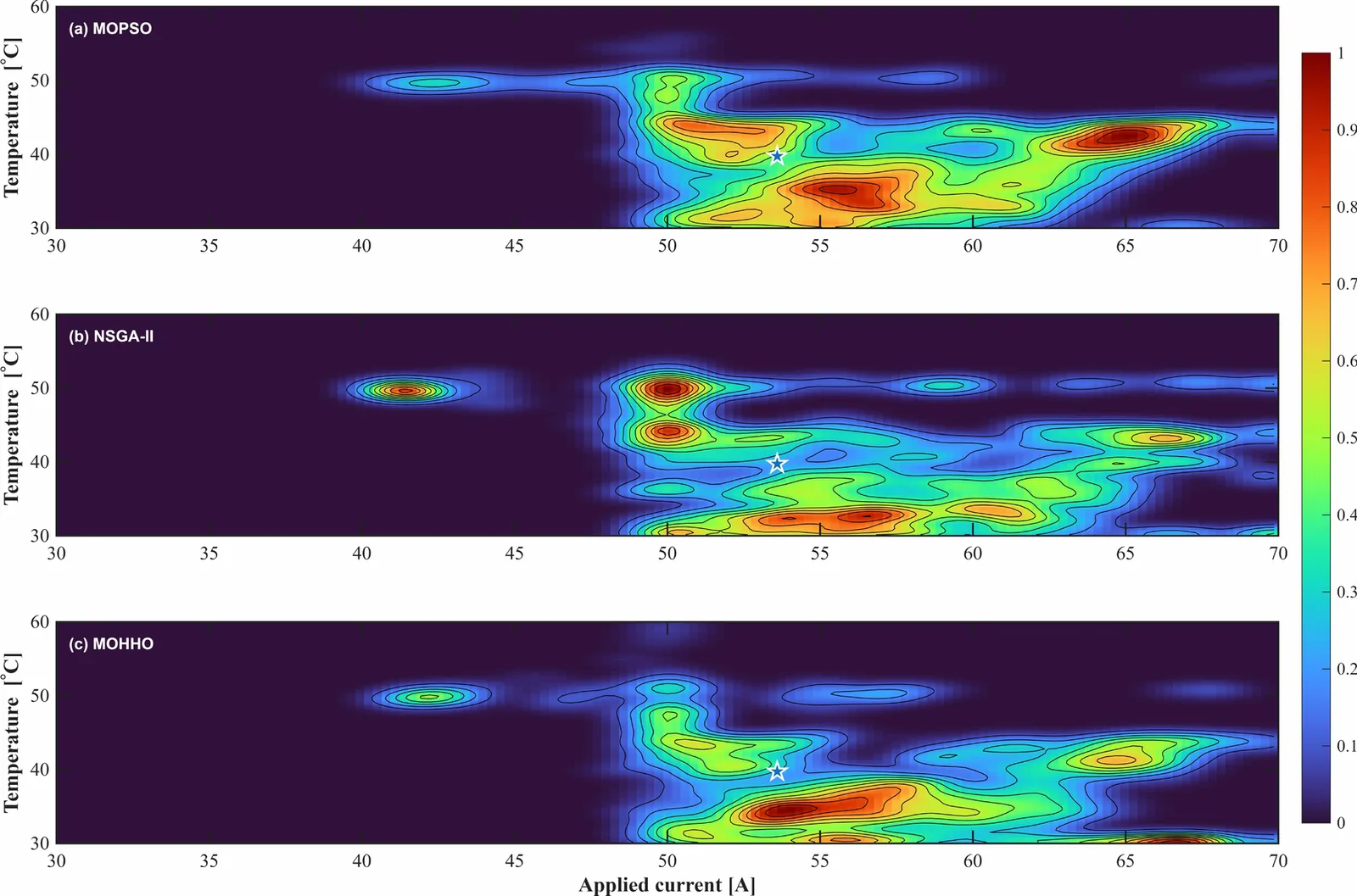
Pareto decision-space density for (**a**) MOPSO, (**b**) NSGA-II and (**c**) MOHHO. Color denotes normalized density of non-dominated current–temperature solutions aggregated over repeated runs. The white star indicates the consensus post-Pareto compromise.

The algorithm-level result is best understood from the density of non-dominated solutions rather than from one scalar endpoint. Figure 2 maps the Pareto decision space for MOPSO, NSGA-II and MOHHO. All three methods identify a favorable region centered broadly around 50–60 A and 35–45 $^{\circ }$C, while their search distributions remain visibly different. The white star marks the consensus post-Pareto compromise near 53.6 A and 39.7 $^{\circ }$C. MOPSO produces a relatively continuous high-density band, NSGA-II maintains several separated non-dominated regions, and MOHHO exhibits a more localized concentration after broader exploration. The consistency of the star location across these different distributions is more informative than forcing each algorithm to report a different “best” point.

**Fig. 3:**
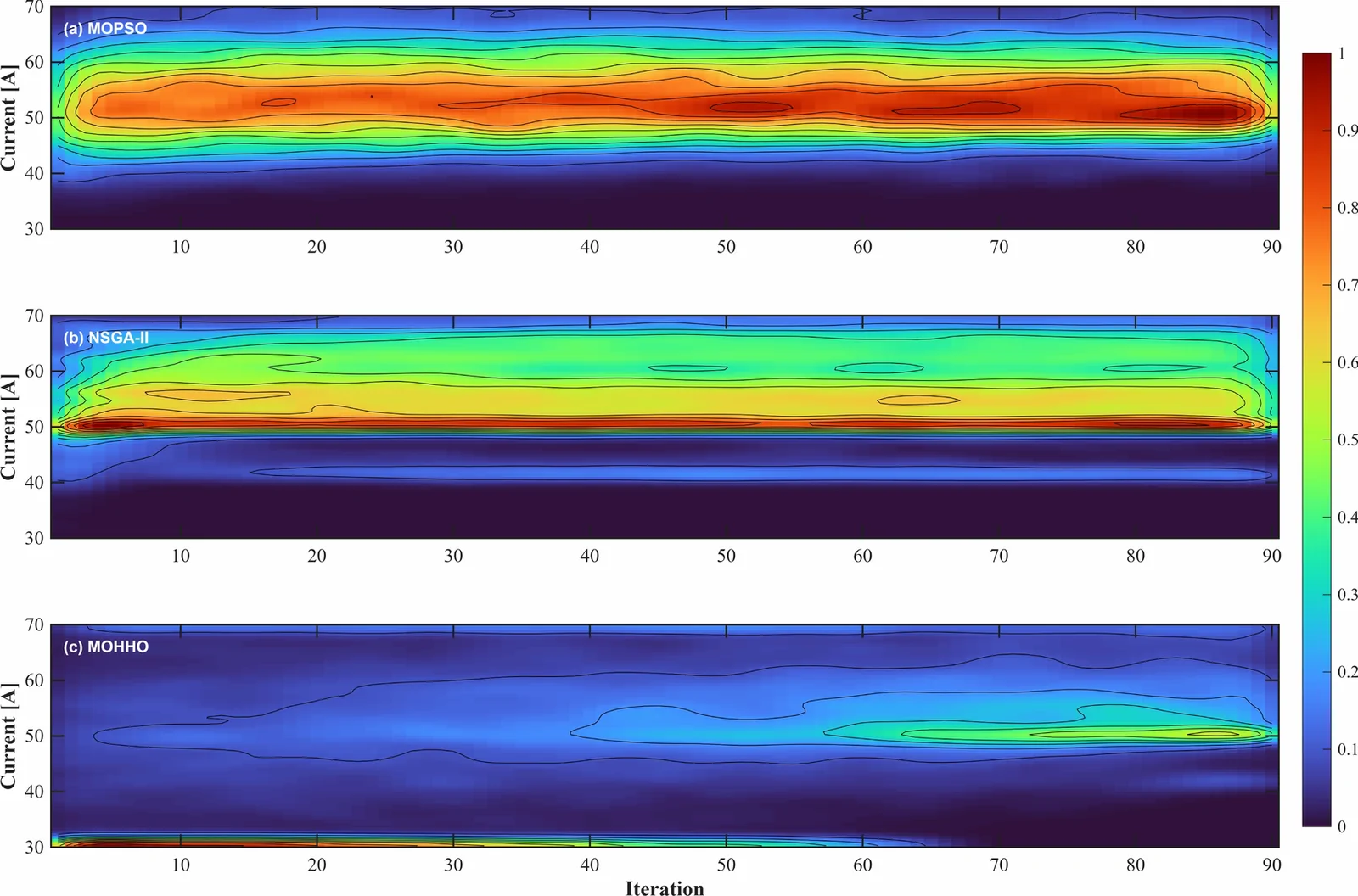
Population search-density maps over iteration for (**a**) MOPSO, (**b**) NSGA-II and (**c**) MOHHO. The plots show algorithmic exploration of applied current and should be interpreted as optimization search-density maps rather than physical energy spectra.

The corresponding population evolution in Fig. 3 explains how the methods reach this region. MOPSO rapidly concentrates around the mid-current band and maintains that band throughout the search. NSGA-II keeps a strong population near 50 A while preserving broader exploration at higher current. MOHHO shows the clearest progressive contraction, with low-current exploration early in the run and stronger concentration near the favorable current band during later iterations. These differences are consistent with the distinct exploration–exploitation mechanisms of the three metaheuristics and support their use as independent checks on the same engineering problem.

### Local robust response and trade-off structure

**Fig. 4:**
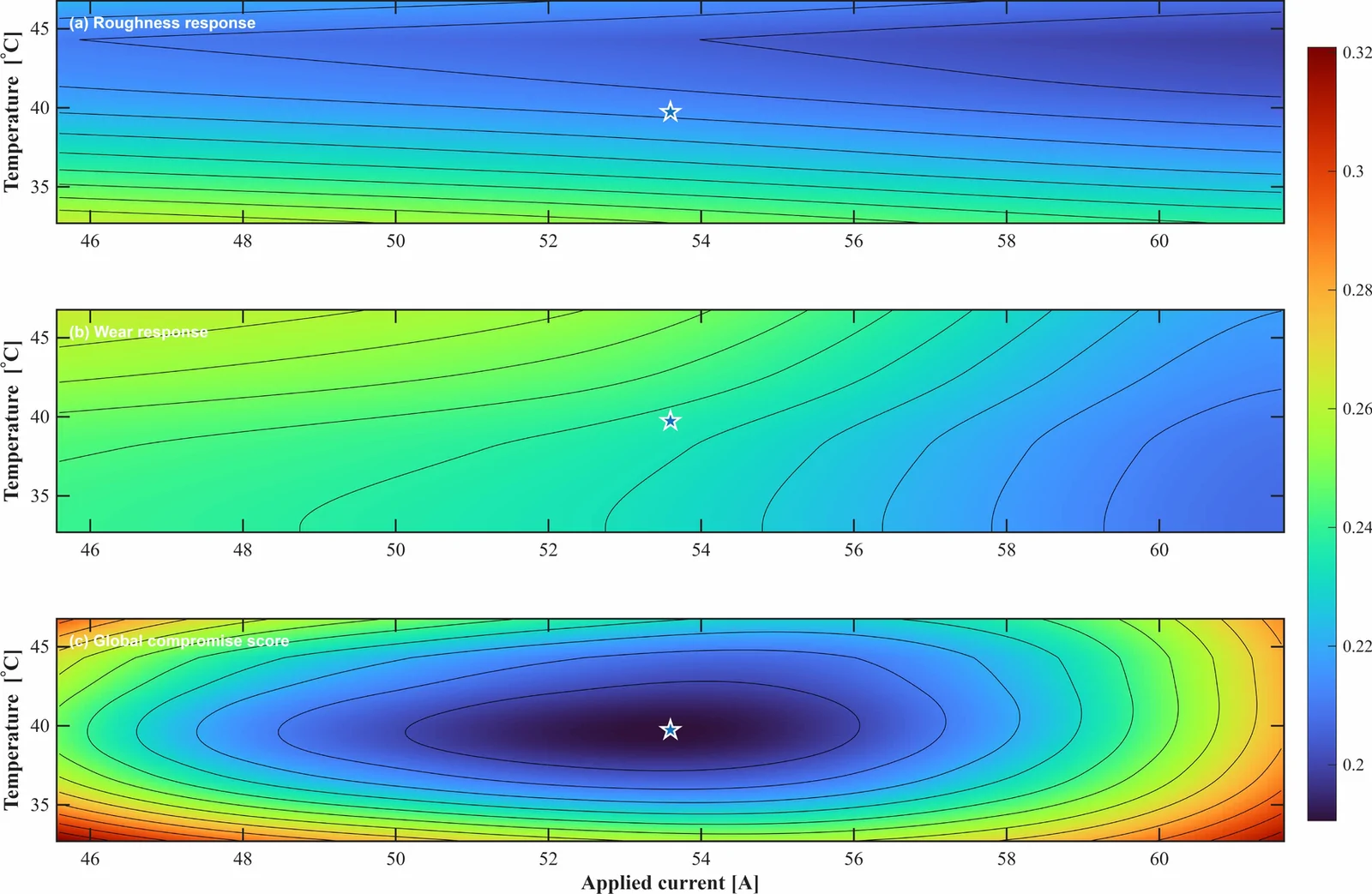
Local robust response maps around the selected operating region: (**a**) normalized roughness response, (**b**) normalized wear response and (**c**) normalized global distance-to-utopia score. The star marks the consensus operating point.

The local response maps in Fig. 4 make the engineering conflict explicit. Roughness decreases toward the upper-temperature side of the local window, while wear shows a different current–temperature gradient. The global compromise score therefore forms a distinct low-value basin rather than simply inheriting the minimum of either individual response. The consensus point at approximately 53.6 A and 39.7 $^{\circ }$C sits near the center of this basin, demonstrating why a multi-objective formulation returns a compromise instead of an extreme single-response condition.

**Fig. 5:**
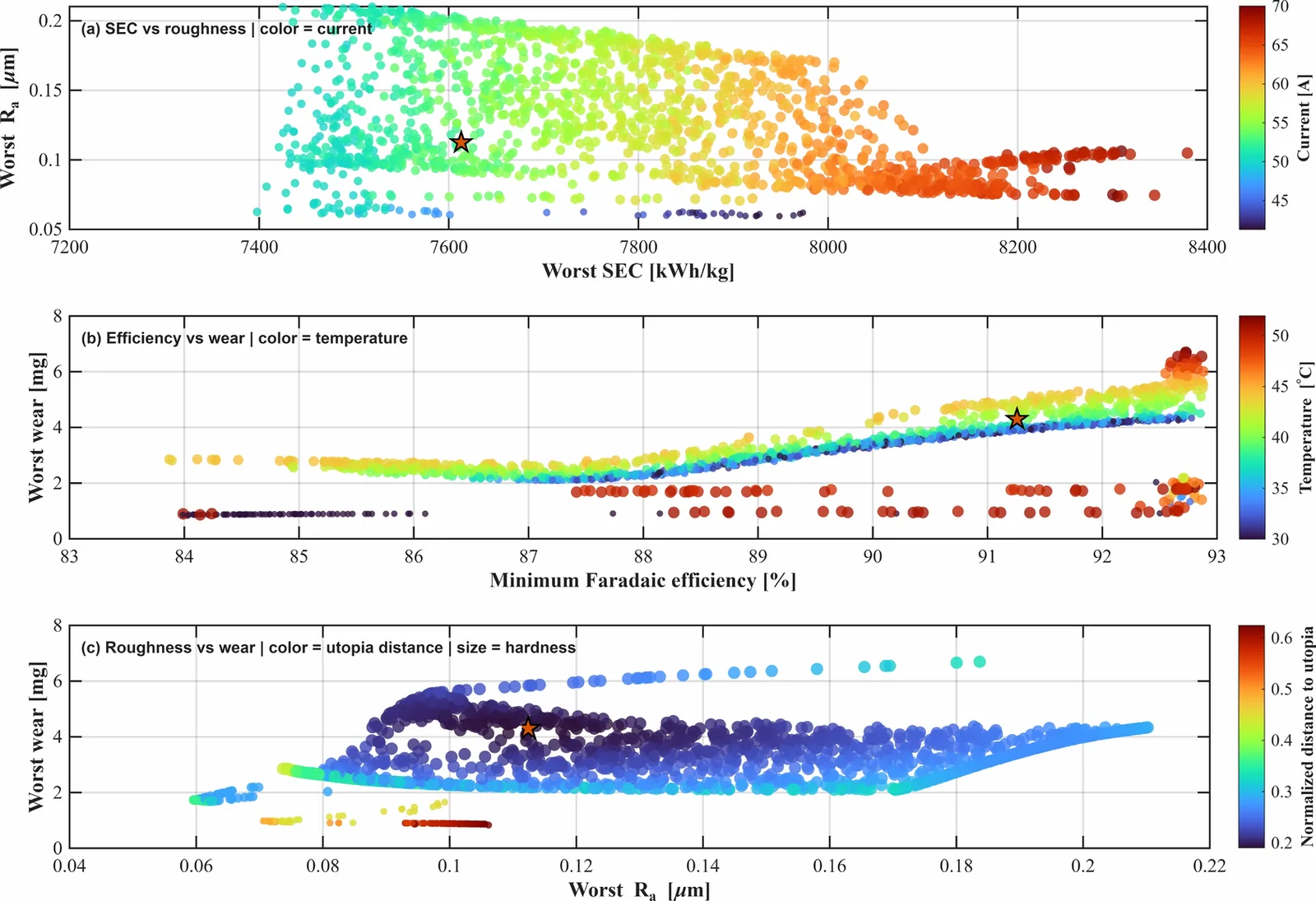
Dense Pareto response clouds. (**a**) Worst SEC versus worst roughness, colored by applied current. (**b**) Minimum Faradaic efficiency versus worst wear, colored by temperature. (**c**) Worst roughness versus worst wear, colored by normalized distance to the utopia point; marker size scales with preparation-level hardness.

The Pareto point clouds in Fig. 5 provide the same trade-offs in response space. In panel (a), the SEC–roughness cloud is colored by current and shows that low SEC does not uniquely correspond to the lowest roughness. Panel (b) reveals that high Faradaic efficiency can coexist with multiple wear levels because temperature and preparation route remain active variables. Panel (c) is the most direct multi-objective view: roughness and wear form several branches associated with different preparation/operating combinations, while color encodes utopia distance and marker size reflects hardness. The selected star is therefore a balanced point on a multi-branch landscape, not the global minimum of roughness or wear separately.

### Baseline comparisons and model-sensitivity checks

Because the decision space contains only two continuous variables and one three-level categorical variable, the metaheuristic results can and should be verified against deterministic baselines; this also answers the legitimate question of whether population-based search is necessary for a problem of this size. Table 5 collects the outcome. An exhaustive grid search with 0.1 A *× * $0.1~^{\circ }$C resolution (362,103 nominal candidates, each evaluated over its 25-point robustness neighbourhood, about $9\times 10^{6}$ response evaluations in total) over the same robust objectives places its minimum-utopia-distance compromise at 53.5 A and 39.7 $^{\circ }$C with diamond polishing, i.e. within the grid resolution of the metaheuristic consensus at 53.6 A and 39.7 $^{\circ }$C. This agreement confirms that the three algorithms converged to the correct region of the model rather than to an artefact of any particular search mechanism. An equal-weight weighted-sum scalarization of the five normalized objectives, minimized over the same grid, selects a nearby but distinct point at 50.1 A and 41.4 $^{\circ }$C; being a linear scalarization it returns a single solution, cannot populate non-convex parts of the front, and gives no access to the multi-branch trade-off structure visible in Fig. 5c. The role of the three metaheuristics in this study is therefore not raw search power on a difficult landscape; it is the generation of well-spread Pareto sets that scalar baselines cannot provide, together with mutual cross-validation of the recommended domain by three independent search mechanisms.

Table 5 also reports the model-sensitivity checks announced in Sect. “Methods”. Replacing the geometric fusion of Eqs. (4), (5) by an arithmetic-mean or an elementwise-maximum rule moves the compromise to 53.1 A/39.3 $^{\circ }$C and 51.6 A/38.0 $^{\circ }$C respectively, in both cases retaining diamond polishing; the recommended operating window is thus stable against the fusion assumption to within about 2 A and 2 $^{\circ }$C. Removing the hardness objective, by contrast, shifts the solution to the electrolytic route at 50.2 A and 50.4 $^{\circ }$C, confirming that the categorical recommendation is controlled by the measured preparation-level hardness advantage, exactly as stated in Sect. “Robust five-objective formulation”.

**Table 5:** Deterministic baselines and model-sensitivity variants of the robust compromise. All variants share the same interpolated responses, robustness neighbourhood and normalization domain; worst-case values refer to the *± 2* A/$\pm 2~^{\circ }$C neighbourhood of each variant’s own compromise.

Variant	$I^{*}$ [A]	$T^{*}$ [$^{\circ }$C]	Route	Worst $R_a$ [*μ *m]	Worst wear [mg]	Worst SEC [kWh kg$^{-1}$]	Min. $\eta _F$ [%]
Metaheuristic consensus (MOPSO/NSGA-II/MOHHO)	53.6	39.7	Diamond	0.113	4.29	7613	91.3
Exhaustive grid search (0.1 A *× * $0.1~^{\circ }$C)	53.5	39.7	Diamond	0.113	4.31	7609	91.3
Equal-weight weighted-sum scalarization	50.1	41.4	Diamond	0.108	5.07	7478	92.7
Arithmetic-mean fusion instead of geometric	53.1	39.3	Diamond	0.117	4.34	7590	91.5
Elementwise-maximum fusion instead of geometric	51.6	38.0	Diamond	0.132	4.40	7526	92.2
Four objectives (hardness term removed)	50.2	50.4	Electrolytic	0.074	0.97	7481	92.7

The new objective-weight sensitivity in Table 6 directly addresses the engineering-preference issue. Increasing only the wear weight from 1 to 2 and 4 keeps diamond polishing but moves the operating point toward higher current, reducing the robust wear from 4.31 mg to 3.61 and 2.74 mg, respectively. At a sixfold wear priority the preferred route switches to 1200-grit polishing (49.3 A, 50.1 $^{\circ }$C; 1.73 mg worst-case wear and 979 HV). If the hardness weight is reduced to 0.25 while the other four weights remain unity, 1200-grit is again selected; if hardness is assigned zero weight, the solution becomes electrolytic polishing at 50.2 A and 50.4 $^{\circ }$C with 0.97 mg worst-case wear and 887 HV. Thus, the reviewer-identified route switch is real and informative: the categorical choice is preference-dependent. The equal-weight diamond solution is not presented as intrinsically superior; it is the reference point associated with equal normalized importance across the five stated objectives.

**Table 6:** Sensitivity of the robust reference compromise to engineering-priority weights. Weights correspond to $[w_{R_a},w_W,w_{SEC},w_{\eta },w_{HV}]$ in Eq. (15). They are illustrative utility scenarios, not experimentally calibrated service-life weights.

Weight scenario	$I^*$ [A]	$T^*$ [$^{\circ }$C]	Route	Worst $R_a$ [*μ *m]	Worst wear [mg]	Min. $\eta _F$ [%]	Mean HV
[1, 1, 1, 1, 1]	53.5	39.7	Diamond	0.113	4.31	91.3	1131
[1, 2, 1, 1, 1]	56.0	38.9	Diamond	0.113	3.61	90.0	1131
[1, 4, 1, 1, 1]	58.9	38.5	Diamond	0.110	2.74	88.6	1131
*[1], [1], [1], [1], [6]*	49.3	50.1	1200-grit	0.064	1.73	92.4	979
[1, 1, 1, 1, 0.25]	49.8	50.1	1200-grit	0.064	1.74	92.6	979
[1, 1, 1, 1, 0]	50.2	50.4	Electrolytic	0.074	0.97	92.7	887

Table 7 then breaks the separability assumption itself. With the adverse non-separable envelope of Eq. (6), *κ =0.25* moves the re-optimized equal-weight reference to 51.9 A/40.3 $^{\circ }$C and gives 0.115 *μ *m worst-case roughness and 4.68 mg worst-case wear. Even the deliberately severe *κ =0.50* envelope retains diamond polishing and a nearby 51.5 A/39.8 $^{\circ }$C reference, with 0.134 *μ *m roughness and 4.79 mg wear. At the original 53.6 A/39.7 $^{\circ }$C point, those two interaction envelopes increase the predicted worst-case roughness by about 8.2% and 22.6%, and wear by about 5.3% and 10.7%, respectively. The stress test therefore does not claim that the separable worst case is an upper physical bound; on the contrary, it demonstrates quantitatively that an unmeasured cross interaction can degrade the predicted values beyond the separable estimate. What remains stable in this deliberately adverse exercise is the broad near-50-A/near-40-$^{\circ }$C decision domain and the equal-weight route selection.

**Table 7:** Non-separable current–temperature interaction stress test. *κ * is the maximum fractional interaction-envelope amplitude in Eq. (6); it is a sensitivity parameter and not a fitted electrochemical coefficient.

Interaction model	$I^*$ [A]	$T^*$ [$^{\circ }$C]	Route	Worst $R_a$ [*μ *m]	Worst wear [mg]	Min. $\eta _F$ [%]
Separable reference, *κ =0*	53.5	39.7	Diamond	0.113	4.31	91.3
Non-separable envelope, *κ =0.25*	51.9	40.3	Diamond	0.115	4.68	92.1
Non-separable envelope, *κ =0.50*	51.5	39.8	Diamond	0.134	4.79	92.2

### Reconstructed roughness profiles and spectral content

**Fig. 6:**
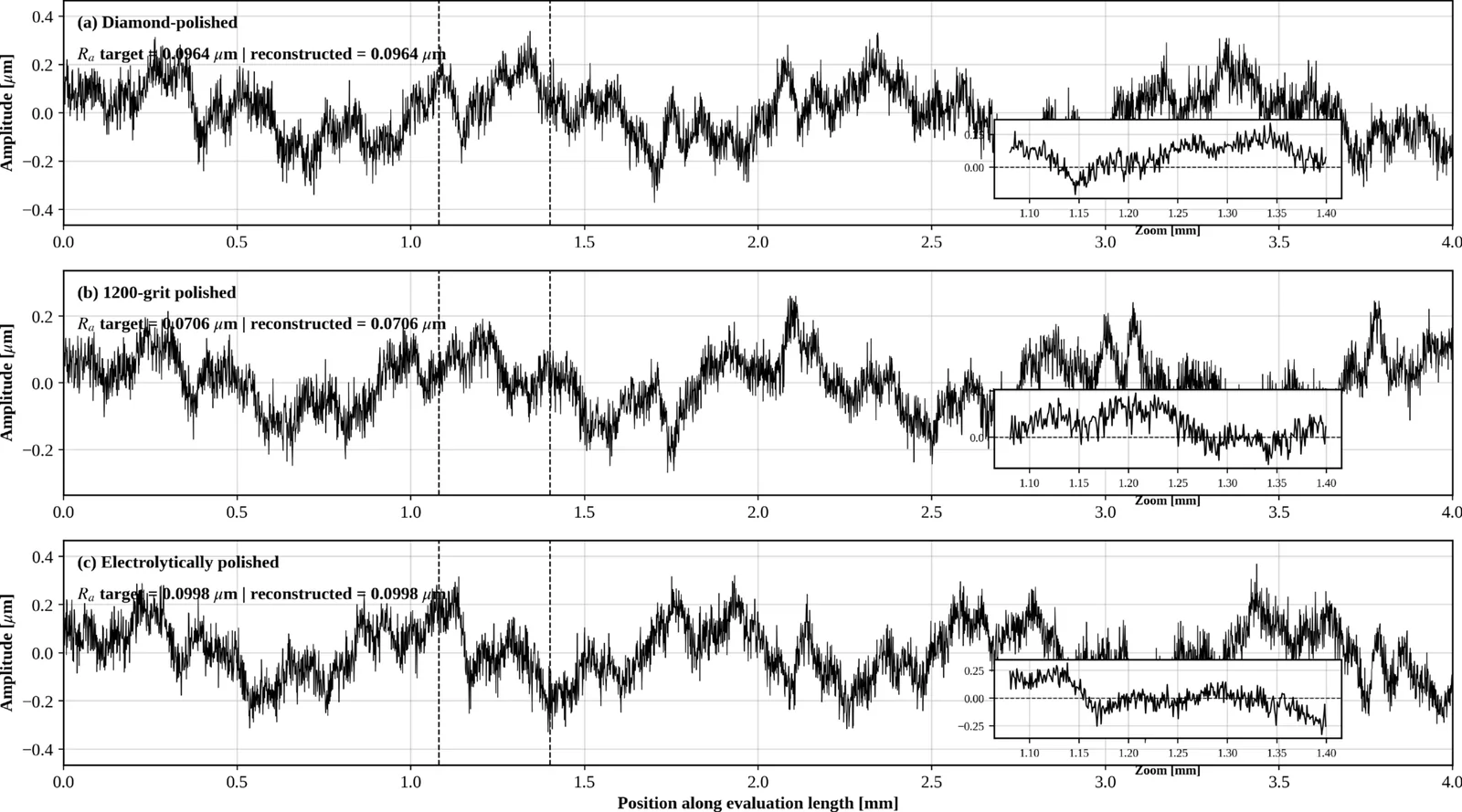
Roughness-consistent reconstructed profiles for (**a**) diamond-polished, (**b**) 1200-grit polished and (**c**) electrolytically polished conditions. Each signal is rescaled so that the calculated arithmetic mean roughness exactly equals the corresponding target $R_a$ at the selected operating condition; inset panels enlarge the marked fluctuation region. The horizontal axes give the physical position along the 4-mm evaluation length sampled at 1-*μ *m intervals, as defined in Sect. “Roughness-consistent reconstructed profiles and PSD”.

Figure 6 translates the three preparation-specific target roughness values at the selected current–temperature condition into roughness-consistent computational profiles. The arithmetic mean roughness calculated from each reconstructed profile exactly matches its target: 0.0964 *μ *m for diamond polishing, 0.0706 *μ *m for 1200-grit polishing and 0.0998 *μ *m for electrolytic polishing. The zoomed windows illustrate how profiles with similar scalar $R_a$ can still contain visibly different local fluctuation patterns. These traces are used only as constrained visual realizations and are not presented as measured profilometer scans. Following the sampling convention introduced in Sect. “Roughness-consistent reconstructed profiles and PSD”, the profiles are now displayed against the physical position along the 4-mm evaluation length.

**Fig. 7:**
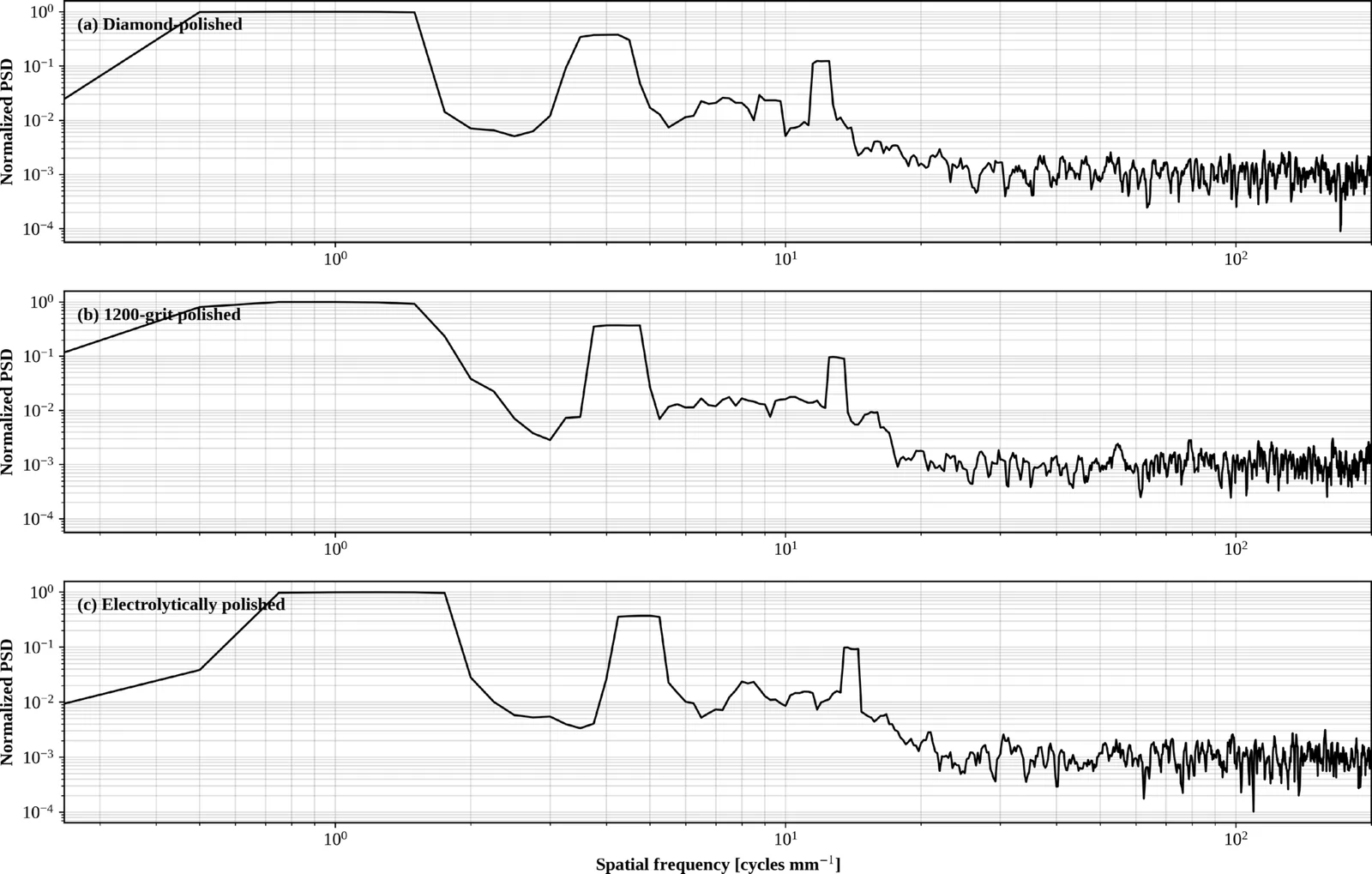
Normalized power spectral density of the reconstructed roughness profiles for (**a**) diamond polishing, (**b**) 1200-grit polishing and (**c**) electrolytic polishing. PSDs are computed directly from the Fig. 6 signals by Welch averaging; the horizontal axis is expressed in cycles per millimetre using the 4-mm/1-*μ *m sampling convention stated in Sect. “Roughness-consistent reconstructed profiles and PSD”.

The PSD representation in Fig. 7 is computed directly from the reconstructed profiles using Welch averaging. With the physical sampling convention of Sect. “Roughness-consistent reconstructed profiles and PSD”, the spectra can now be read in inverse-length units. All three routes are dominated by long-wavelength content between roughly 0.7 and 1.5 cycles mm$^{-1}$, corresponding to wavelengths of about 0.7–1.3 mm, with secondary components near 4–5 cycles mm$^{-1}$ and 12–14 cycles mm$^{-1}$ inherited from the deterministic waviness terms of the reconstruction, and a broadband floor beyond about 20 cycles mm$^{-1}$. The value of the figure lies in comparing the frequency distribution of the constrained reconstructions, not in claiming experimentally measured surface wavelengths; the quoted wavelengths should be validated against stylus or optical profilometry with a documented instrument pitch before being used quantitatively.

## Discussion

The most important result is not that one metaheuristic returns a numerically different optimum from the others. On this low-dimensional engineering problem, competent optimizers should often converge to overlapping Pareto regions. The relevant comparison is therefore whether independent algorithms repeatedly concentrate around the same favorable decision domain while preserving different distributions of non-dominated solutions. Figures 2 and 3 show exactly that behaviour. MOPSO, NSGA-II and MOHHO differ in how they explore current and temperature, yet all support a compromise around the mid-current, near-40 $^{\circ }$C region.

This consensus is physically reasonable when the source data are considered. The current-energy series favours 50 A, where the supplied Faradaic efficiency reaches 93% and SEC reaches its minimum. Roughness and wear, however, do not share that same optimum. Diamond polishing offers the highest preparation-level hardness, electrolytic polishing offers very low wear in parts of the temperature sweep, and 1200-grit polishing provides the smallest measured roughness at 50 $^{\circ }$C. The robust Pareto solution selects diamond polishing because the hardness objective gives it a substantial mechanical advantage while the current and temperature variables move away from conditions where roughness, wear or energy penalties become severe. The result should therefore be interpreted as the optimum of the stated five-objective model, not as proof that diamond polishing is universally superior for every service requirement.

The use of a deterministic *± 2* A/$\pm 2~^{\circ }$C neighbourhood is also important. A single interpolated point can look artificially attractive when experimental sampling is sparse. Evaluating 25 local perturbations makes the objective sensitive to nearby degradation and discourages solutions located on narrow numerical minima. The approach is a robustness stress test rather than a statistical uncertainty analysis; no probability distribution or repeated-measurement variance is inferred from the perturbation grid.

The geometric reconstruction in Eqs. (4), (5) is the principal methodological limitation and should be interpreted transparently. It exists because the original experiments contain one current sweep at fixed temperature and one temperature sweep at fixed current, rather than a full factorial matrix. The reconstruction provides a mathematically smooth way to combine those orthogonal trends for post-experimental optimization, but it cannot reproduce unknown current–temperature interaction effects. A future experimental design should therefore measure a factorial or response-surface matrix around the Pareto basin, especially near 50–60 A and 35–45 $^{\circ }$C, and should include replicates so that confidence intervals and analysis of variance can replace the present deterministic stress test.

Accordingly, the current manuscript does not claim that a true coupled electrochemical fluctuation is bounded by the original 25-point separable robustness values. Such a guarantee is impossible from two orthogonal sweeps alone. The non-separable envelope in Eq. (6) is included precisely to avoid a self-validating robustness argument: it preserves every measured sweep point while allowing a cross term that changes the response away from those lines. The resulting 25–50% interaction-envelope tests show that the numerical worst cases can indeed rise beyond the separable prediction, while the preferred equal-weight region remains nearby. This is evidence of decision-domain sensitivity, not identification of the true interaction physics. Only a factorial experiment with replicated *I*–*T* combinations can estimate the sign and magnitude of the coupling, permit an interaction term to be fitted, and provide a defensible probabilistic robustness bound.

The same distinction applies to Figs. 6 and 7. A scalar $R_a$ value does not uniquely determine a surface profile or PSD. The reconstructed signals are constrained to match the target arithmetic roughness and are useful for visualizing one possible distribution of surface fluctuations consistent with that scalar level, but they are not unique and should never be described as direct profilometry. Real surface-topography validation requires raw stylus or optical profilometry with known sampling pitch, scan length, filtering standard and repeated traces. The current PSD plots therefore remain interpretive computational views.

From a coating-engineering perspective, the selected condition does not remove the need for microstructural validation. Chromium-coating wear can depend on microcrack density^11^, oxidation and structural evolution^16^, coating architecture^18^ and third-body or transfer-film behaviour^25^. Cross-sectional coating thickness, crack density, residual stress, adhesion, SEM/EDS of wear tracks, debris chemistry and long-duration friction evolution are therefore necessary before the post-experimental optimum can be generalized to service life.

A specific point concerns the wear mechanism at the high preparation-level hardness of about 1131 HV. Electrodeposited chromium at this hardness level frequently exhibits high brittleness and a network of microcracks, and under repeated sliding contact material can then be removed by crack growth and fatigue delamination rather than by mild abrasion^6,11,17^. The load dependence of the present hardness data points in the same direction: the diamond-route hardness decreases from 1350 HV at 25 g to 850 HV at 1000 g, a drop that exceeds a pure indentation-size effect and is consistent with subsurface cracking being activated at higher indentation loads. Mass loss alone cannot discriminate between abrasive, adhesive, oxidative and fatigue-delamination contributions, so the wear objective $f_2$ must be read as an integral severity measure and not as evidence of a particular mechanism. SEM/EDS examination of the wear tracks and debris, cross-sectional imaging of subsurface cracking and friction-evolution records at the recommended condition are required before mechanistic conclusions can be attached to the optimized point; these observations are part of the confirmation campaign described below.

The energy interpretation requires similar caution. The absolute SEC values are retained from the supplied laboratory series because they provide a consistent current-dependent penalty for optimization. They should not be treated as a verified industrial energy footprint until cell-voltage history, deposited mass, bath-heating demand and deposition time are reported with traceable units and uncertainty. The value of the present energy term is comparative: it prevents the optimizer from selecting a mechanically attractive condition at an obviously unfavorable electrical setting.

**Fig. 8:**
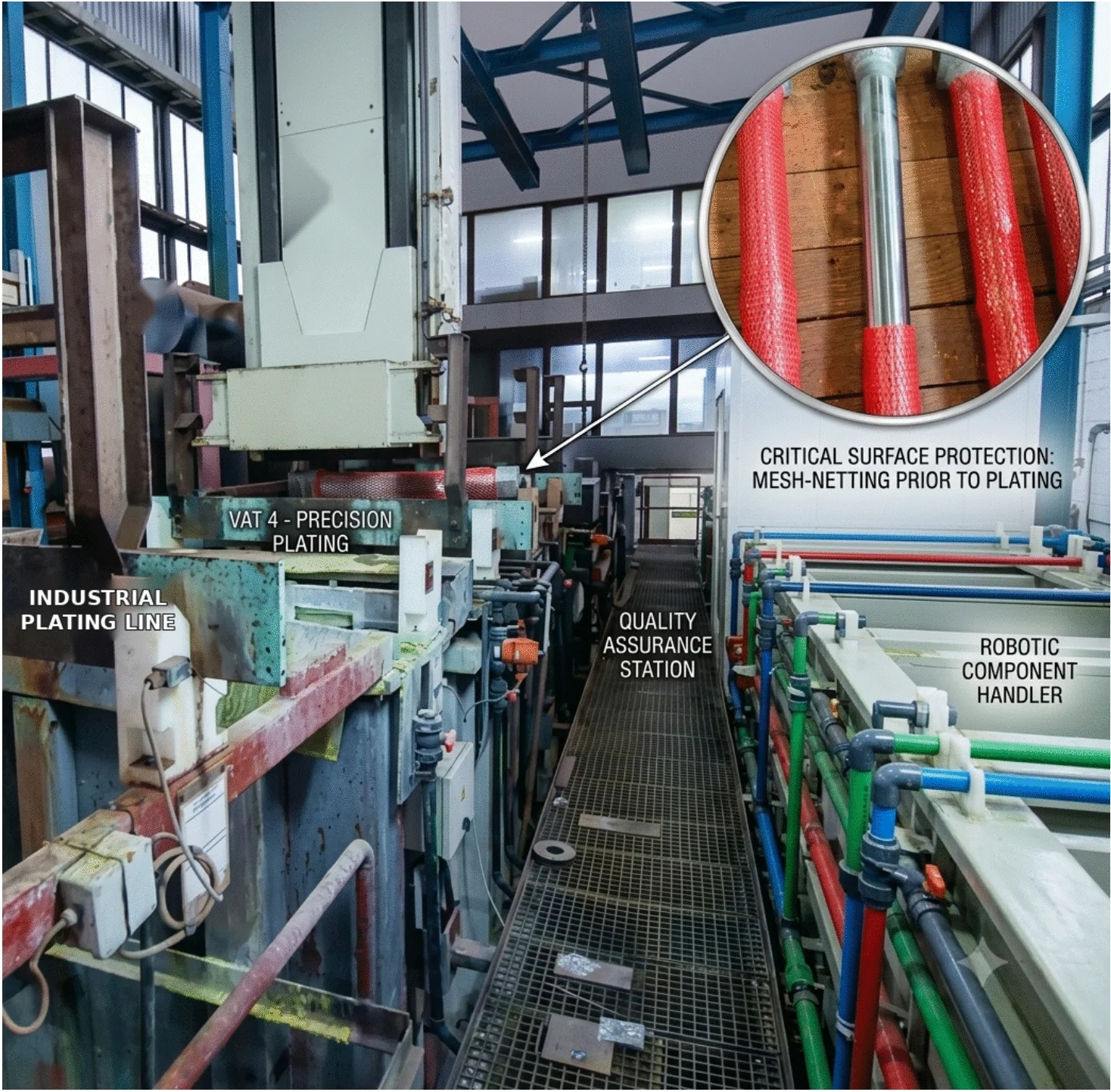
Industrial chromium-plating context included as an application illustration. Translation of the identified Pareto window to production would require current-density normalization by active area, bath-chemistry control, voltage and thermal-energy logging, coating-thickness verification and long-duration quality assurance.

The industrial context is illustrated in Fig. 8. Large-scale plating lines involve component masking, bath control, handling and quality assurance that are absent from a small laboratory response dataset. Accordingly, the computational result is best viewed as a candidate process window to be validated experimentally before transfer to production.

In terms of everyday plating practice, the recommended point is nevertheless well placed. With the 1.0 dm$^{2}$ specimen area, 53.6 A corresponds to a mean cathodic current density of about 53.6 A dm$^{-2}$, which lies inside the 30–60 A dm$^{-2}$ window commonly applied in industrial hard-chromium deposition, so the condition can be realized on standard rectifiers without unusual demands. The recommended bath temperature near 40 $^{\circ }$C is 10–20 $^{\circ }$C lower than the 50–60 $^{\circ }$C typically maintained in hexavalent hard-chromium tanks, which reduces bath-heating demand and evaporation losses at production scale. Just as importantly, the compromise was selected under *± 2* A and $\pm 2~^{\circ }$C perturbations, which is precisely the order of setpoint tolerance that industrial current and temperature controllers are expected to hold; an operator can therefore run the condition as a practical setpoint of 54 A (*≈ *54 A dm$^{-2}$) and 40 $^{\circ }$C without the predicted performance depending on unrealistically tight control.

Finally, the experimental status of the recommended condition must be stated without ambiguity. No new specimens were deposited at 53.6 A and 39.7 $^{\circ }$C during the current response period, because the plating and characterization facilities of the original campaign were not available within that period; every value quoted at that condition is a reconstruction from the measured sweeps, and the manuscript deliberately marks it as such throughout. Two anchoring observations nevertheless bound the prediction. First, the recommended point lies only 3.6 A and 0.3 $^{\circ }$C away from the measured condition of 50 A and 40 $^{\circ }$C, where the diamond route gave measured roughness of 0.080–0.130 *μ *m and wear of 4.30–4.40 mg in the two sweeps, together with the highest measured efficiency of the series (93%) and its lowest SEC; the nominal reconstructed responses at the compromise (0.096 *μ *m, 3.63 mg, 92.2%) are consistent with these experimental brackets. Second, the deterministic baselines of Sect. “Baseline comparisons and model-sensitivity checks” confirm that the point is not an artefact of any single algorithm. A dedicated confirmation campaign, consisting of replicated depositions at the recommended setpoint and at two flanking conditions with measured roughness, wear, hardness, coating thickness, cathodic efficiency and logged cell voltage, is the planned next step; the framework presented here should be read as the decision-support stage that designs that campaign, not as its substitute.

## Conclusion

This study reformulates a Cr(III) electrodeposition dataset for 42CrMo4 steel as a robust five-objective Pareto problem instead of selecting an operating condition from individual experimental curves. Surface roughness, wear, SEC, Faradaic efficiency and preparation-level hardness are optimized simultaneously using MOPSO, NSGA-II and MOHHO over current, temperature and surface preparation. The three algorithms generate different search-density patterns but identify a common favorable region, supporting a consensus compromise near 53.6 A and 39.7 $^{\circ }$C with diamond polishing under the stated model. With the reported effective plated area of 1.0 dm$^{2}$, this current corresponds to a mean cathodic current density of about 53.6 A dm$^{-2}$, inside the standard industrial hard-chromium window. The deterministic grid-search and weighted-sum baselines added in the current manuscript reproduce the same operating region, and the sensitivity checks show that the region is stable against the fusion assumption, while the preparation-route choice is governed by the hardness objective.

At the selected point, the local robustness test gives a worst-case reconstructed roughness of about 0.112 *μ *m, worst-case wear of about 4.29 mg and minimum Faradaic efficiency of about 91.3%. The preparation-level hardness index remains about 1131 HV. These values should be interpreted as the outcome of the explicit multi-objective formulation: the chosen point is not the minimum of roughness, wear or energy separately, but a compromise that remains favorable over a *± 2* A/$\pm 2~^{\circ }$C neighbourhood.

The work also clarifies the boundary between measurement and computation. The current and temperature sweeps, hardness data, efficiency trend and SEC trend are experimental inputs; the two-dimensional *I*–*T* maps are geometric reconstructions of orthogonal sweeps; and the roughness profiles and PSDs are computational realizations constrained by target $R_a$. This separation is essential for reproducibility and prevents post-experimental visualization from being mistaken for new measurement.

The new sensitivity analysis also changes how the recommended point should be read. The diamond route is the equal-weight reference choice, but route selection shifts toward 1200-grit or electrolytic polishing when wear is prioritized or hardness is de-emphasized. Moreover, an explicitly non-separable *I*–*T* interaction envelope can increase the predicted worst-case roughness and wear beyond the original separable robustness values. These results make the engineering preference structure and the interaction-model limitation explicit rather than hiding them inside a single scalar compromise.

Future work should experimentally sample the identified Pareto basin with a factorial design, continuously log cell voltage and bath-heating power, measure deposited mass and coating thickness, and add replicated roughness, wear and microhardness tests. SEM/EDS, residual stress, adhesion, crack-density measurements and long-duration tribology would then allow the present Pareto framework to move from a post-experimental decision aid to a validated process-qualification tool.

## Supplementary Information

Supplementary Information.

## Data Availability

The numerical source values used in the post-experimental optimization are reported in the manuscript and are included in the accompanying MATLAB source package. The reconstructed response surfaces and profile spectra are generated deterministically from those values. Additional raw experimental records, where available, may be requested from the corresponding author.

## Code availability

The MATLAB scripts used to generate the multi-objective figures and the corrected Welch-PSD representation are included in the accompanying source package. The current source package additionally contains the deterministic grid-search and weighted-sum baseline computations and the fusion-rule and hardness-objective sensitivity checks reported in Sect. “Baseline comparisons and model-sensitivity checks”. For the current response round, the code additionally includes the weighted normalized-distance scenarios of Eq. (15) and the non-separable interaction-envelope stress test of Eq. (6).
